# Impact of Baseline Central Retinal Thickness on Outcomes in the VIVID-DME and VISTA-DME Studies

**DOI:** 10.1155/2018/3640135

**Published:** 2018-03-29

**Authors:** Edoardo Midena, Mark Gillies, Todd A. Katz, Carola Metzig, Chengxing Lu, Yuichiro Ogura

**Affiliations:** ^1^Department of Ophthalmology, University of Padova, Padua, Italy; ^2^Save Sight Institute, Sydney Medical School, University of Sydney, Sydney Eye Hospital, Sydney, NSW, Australia; ^3^Bayer US, LLC, Whippany, NJ, USA; ^4^Bayer AG, Berlin, Germany; ^5^Department of Ophthalmology & Visual Science, Nagoya City University Graduate School of Medical Sciences, Nagoya, Japan

## Abstract

**Purpose:**

To report the impact of baseline central retinal thickness (CRT) on outcomes in patients with diabetic macular edema (DME) in VIVID-DME and VISTA-DME.

**Methods:**

Post hoc analyses of two randomized controlled trials in which 862 DME patients were randomized 1 : 1 : 1 to treatment with intravitreal aflibercept 2.0 mg every 4 weeks (2q4), intravitreal aflibercept 2.0 mg every 8 weeks after five initial monthly doses (2q8), or macular laser photocoagulation at baseline and as needed. We compared visual and anatomical outcomes in subgroups of patients with baseline CRT < 400 *μ*m and ≥400 *μ*m.

**Results:**

At weeks 52 and 100, outcomes with intravitreal aflibercept 2q4 and 2q8 were superior to those in laser control-treated patients regardless of baseline CRT. When looked at in a binary fashion, the treatment effect of intravitreal aflibercept versus laser was not significantly better in the ≥400 *μ*m than the <400 *μ*m group; when looked at as a continuous variable, baseline CRT seemed to have an impact on the treatment effect of intravitreal aflibercept versus laser.

**Conclusions:**

Post hoc analyses of VIVID-DME and VISTA-DME demonstrated the benefits of intravitreal aflibercept treatment in DME patients with baseline CRT < 400 *μ*m and ≥400 *μ*m. This trial is registered with NCT01331681 and NCT01363440.

## 1. Introduction

Diabetic retinopathy (DR), the most common microvascular complication in patients with diabetes mellitus, is the leading cause of blindness in working-age adults [1, 2]. The vision loss associated with DR is caused in large part by diabetic macular edema (DME) [1, 2] and can occur at any stage of DR. The estimated global prevalence of DME is currently around 21 million cases [3]; this is expected to increase with the rising prevalence of diabetes, which is projected to affect up to 592 million people worldwide by 2035 [2].

Treatment with antivascular endothelial growth factor (VEGF) drugs has increasingly replaced laser photocoagulation as the standard of care in DME. Both ranibizumab and intravitreal bevacizumab have demonstrated efficacy and safety in the treatment of DME [4–8], although bevacizumab is not licensed for ophthalmic use. The VIVID-DME and VISTA-DME studies showed superior visual and anatomical outcomes with intravitreal aflibercept monotherapy compared with laser monotherapy [9–11]. At its primary endpoint, the recent Protocol T study conducted by the Diabetic Retinopathy Clinical Research Network (http://DRCR.net) demonstrated statistical superiority of intravitreal aflibercept over ranibizumab or bevacizumab at 12 months, particularly in patients with baseline visual acuity of 20/50 or worse [12]. Improvement in VA at the 2-year time point with intravitreal aflibercept remained statistically superior to bevacizumab but not ranibizumab, and an area under the curve analysis showed that the mean change in visual acuity over 2 years was greater with intravitreal aflibercept than with bevacizumab or ranibizumab [13].

The impact of baseline central retinal thickness (CRT) and best-corrected visual acuity (BCVA) has been analyzed in several studies of ranibizumab in patients with DME. Subanalyses of the RESTORE [14] and Protocol I [15] studies found that patients with thicker retinas at baseline experienced greater gains in vision compared with patients with thinner retinas. Based on guidance from the National Institute for Health and Care Excellence (NICE), reimbursement for anti-VEGF therapy for DME in the United Kingdom is limited to patients with CRT of ≥400 *μ*m at the start of treatment [16].

Another subanalysis of the RESTORE study showed that patients with DME who had poorer baseline visual acuity achieved greater gains than those with better baseline vision [17]. The impact of baseline BCVA on visual outcomes in other retinal disorders has also been evaluated. In the “Comparison of Age-Related Macular Degeneration Treatments Trials” in patients with neovascular age-related macular degeneration, better baseline visual acuity was associated with less gain in visual acuity [18], and a meta-analysis of studies in age-related macular degeneration demonstrated that worse visual acuity at baseline predicted more gains in vision [19].

Since there are currently no data on the impact of CRT at baseline on outcomes with intravitreal aflibercept compared with laser control, we decided to test the hypothesis that resulted from the above evidence by analyzing the impact of this baseline factor on visual and anatomical outcomes in patients with DME enrolled in the VIVID-DME and VISTA-DME studies and, specifically, to test the NICE guidance that would disallow reimbursement for covered patients with thinner retinas (<400 *μ*m) from receiving intravitreal aflibercept.

## 2. Patients and Methods

### 2.1. Design

The study design and methods have been published previously [9–11]. Key details are summarized here. VIVID-DME (NCT01331681) and VISTA-DME (NCT01363440) were phase III, randomized, double-masked, active-controlled, 148-week trials comparing two dosing regimens of intravitreal aflibercept with laser control for the treatment of DME. The studies were conducted at 127 sites in the United States, Europe, Japan, and Australia and were conducted in accordance with the principles of the Health Insurance Portability and Accountability Act, the Declaration of Helsinki, and the International Conference on Harmonisation. Institutional review board/ethics committee approval was obtained at each site before the start of the studies. All patients signed a written consent form before the initiation of study-specific procedures. Patients in the laser control treatment group were eligible to receive intravitreal aflibercept treatment in the third year of the studies; therefore, only week 52 and week 100 data are included in these post hoc analyses.

### 2.2. Patients

Adult patients with type 1 or type 2 diabetes mellitus who presented with DME with central involvement (defined as retinal thickening involving the 1 mm central subfield) were included if BCVA was between 73 and 24 letters (20/40–20/320 Snellen equivalent) in the study eye. Only one eye per patient was included.

### 2.3. Randomization and Treatment

Patients were randomized 1 : 1 : 1 to treatment with intravitreal aflibercept 2.0 mg every 4 weeks (2q4), intravitreal aflibercept 2.0 mg every 8 weeks after five initial monthly doses (2q8), or macular laser photocoagulation at baseline. To preserve masking, the eyes in the laser control group received sham injections at every visit and the eyes in the 2q8 group received sham injections on nontreatment visits. From week 24, additional active treatment (laser in the intravitreal aflibercept groups and intravitreal aflibercept in the laser control group) was allowed in the case of disease recurrence/worsening based on prespecified criteria.

### 2.4. Outcomes

The primary efficacy endpoint for the VIVID-DME and VISTA-DME studies was the change from baseline in BCVA in “Early Treatment Diabetic Retinopathy Study” (ETDRS) letters at week 52.

Here, we report on the impact of baseline CRT (<400 *μ*m or ≥400 *μ*m, which mirrors the cut-offs used in the NICE reimbursement guidelines) on outcomes in patients enrolled in VIVID-DME and VISTA-DME. The additional impact of baseline BCVA (<40, ≥40 to <55, ≥55 to <65, and ≥65 ETDRS letters) was also assessed within the two baseline CRT groups. Data from the two studies have been integrated.

### 2.5. Statistics

Patients included in the efficacy analyses were those from the full analysis set (FAS) in both studies (VIVID-DME and VISTA-DME), which included all randomized patients who received any study medication and had at least one baseline and one postbaseline assessment. The FAS was analyzed as randomized. Baseline CRT was dichotomized into two subgroups, <400 *μ*m and ≥400 *μ*m. For continuous endpoints such as change from baseline in BCVA and change from baseline in CRT, an analysis of covariance (ANCOVA) model was fitted with baseline BCVA, dichotomized baseline CRT (<400 *μ*m and ≥400 *μ*m), treatment group, study, and interaction between dichotomized baseline CRT and treatment as the fixed effect. Nominal *P* values were presented in these ad hoc analyses without further multiplicity adjustment. As sensitivity analyses, dichotomized baseline CRT was replaced by the continuous baseline CRT in the previously described models to explore whether a significant treatment effect was seen for various continuous baseline CRT values. For binary endpoints, such as the proportion of patients who gained or lost ≥10 and ≥15 letters, the counts and percentages were calculated for each treatment group, with the treatment difference along with the Cochran-Mantel-Haenszel 95% confidence interval adjusted by study for each subgroup. Nominal *P* values were also calculated from the Cochran-Mantel-Haenszel test without further multiplicity adjustments. Missing values in the outcomes were imputed using the last observation carried forward method, and for the eyes that received additional treatment, the last value before additional treatment was used for analysis.

Patients included in the safety analyses were those from the safety population in both studies, which included all randomized patients who received any study treatment.

Selection of patient populations and imputation of missing values were conducted as was done by Staurenghi et al. (2017).

## 3. Results

Baseline demographics and disease characteristics of patient subgroups with baseline CRT < 400 *μ*m (*n* = 246) or ≥400 *μ*m (*n* = 616) are reported in Table 1. Mean baseline visual acuity was lower among patients in the ≥400 *μ*m baseline CRT subgroup, and the distribution of patients across baseline BCVA categories and diabetic retinopathy severity scale (DRSS) scores differed between the baseline CRT subgroups (i.e., more severe baseline BCVA and DRSS scores were more likely to be in the subgroup with thicker retinas at baseline).

**Figure 1:**
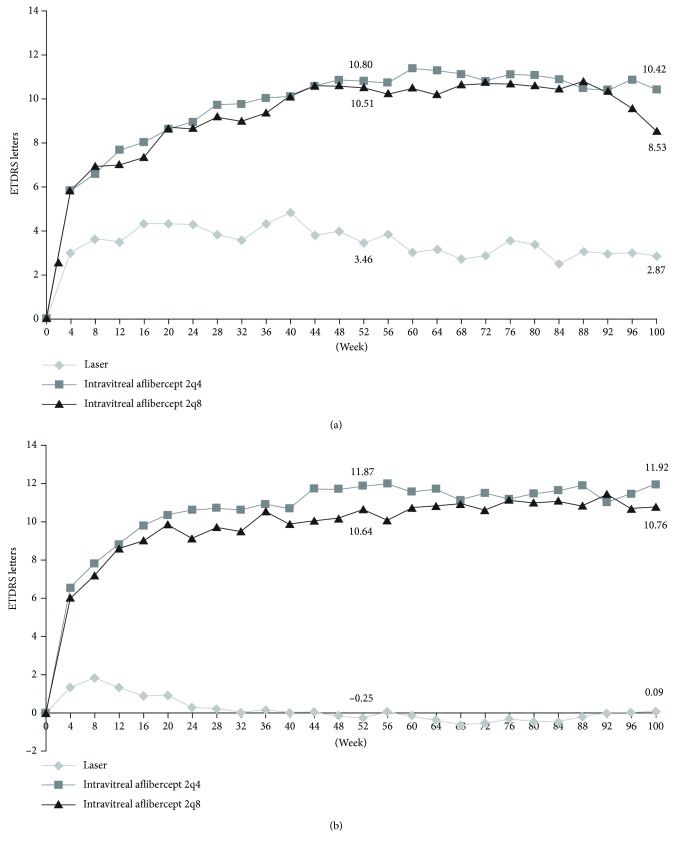
Least squares mean change in best-corrected visual acuity by a visit from baseline to week 100 for patients with (a) baseline central retinal thickness < 400 *μ*m and (b) baseline central retinal thickness ≥ 400 *μ*m (full analysis set, last observation carried forward, adjusted for baseline visual acuity). ETDRS: Early Treatment Diabetic Retinopathy Study.

For all visual and anatomical outcomes assessed in this analysis, intravitreal aflibercept was superior to laser, regardless of baseline CRT. After the adjustment for baseline BCVA, the least squares mean gain in BCVA was greater among patients treated with intravitreal aflibercept (both 2q4 and 2q8) at week 52 (Table 2) and week 100 compared with laser control-treated patients (Figure 1, Table 3). Likewise, the mean decrease in CRT was greater among patients treated with intravitreal aflibercept 2q4 and 2q8 at week 52 (Table 2) and week 100 (Table 3) compared with laser control-treated patients.

Irrespective of baseline CRT, the proportion of patients who gained ≥10 or ≥15 ETDRS letters at week 52 and week 100 was greater among patients treated with intravitreal aflibercept 2q4 and 2q8 than among those treated with laser control. Conversely, in both studies, a smaller proportion of patients in the intravitreal aflibercept 2q4 and 2q8 groups lost ≥10 or ≥15 ETDRS letters at week 52 (Table 2) and week 100 (Table 3) compared with patients in the laser control group.

Regardless of baseline CRT, the proportion of patients treated with intravitreal aflibercept 2q4 and 2q8 versus laser control who experienced a ≥2-step improvement in the DRSS score at week 52 (Table 2) and week 100 (Table 3) was greater than the proportion of laser control-treated patients who experienced such an improvement.

The treatment effect of intravitreal aflibercept was numerically slightly smaller in the group with baseline CRT < 400 *μ*m than in the group with baseline CRT ≥ 400 *μ*m. A nominal test of interactions in an ad hoc fashion with binary baseline CRT failed to show evidence that the treatment effect of intravitreal aflibercept versus laser control was statistically significantly different between the two baseline CRT subgroups (*P* = 0.0637). When considered a continuous variable, there may be some impact of baseline CRT on the treatment effect through week 100 in terms of mean change in BCVA (nominal *P* value for interaction is equal to 0.0637); however, this impact was not seen for the proportion of patients who gained ≥10 or ≥15 letters or for DRSS outcomes.

**Figure 2:**
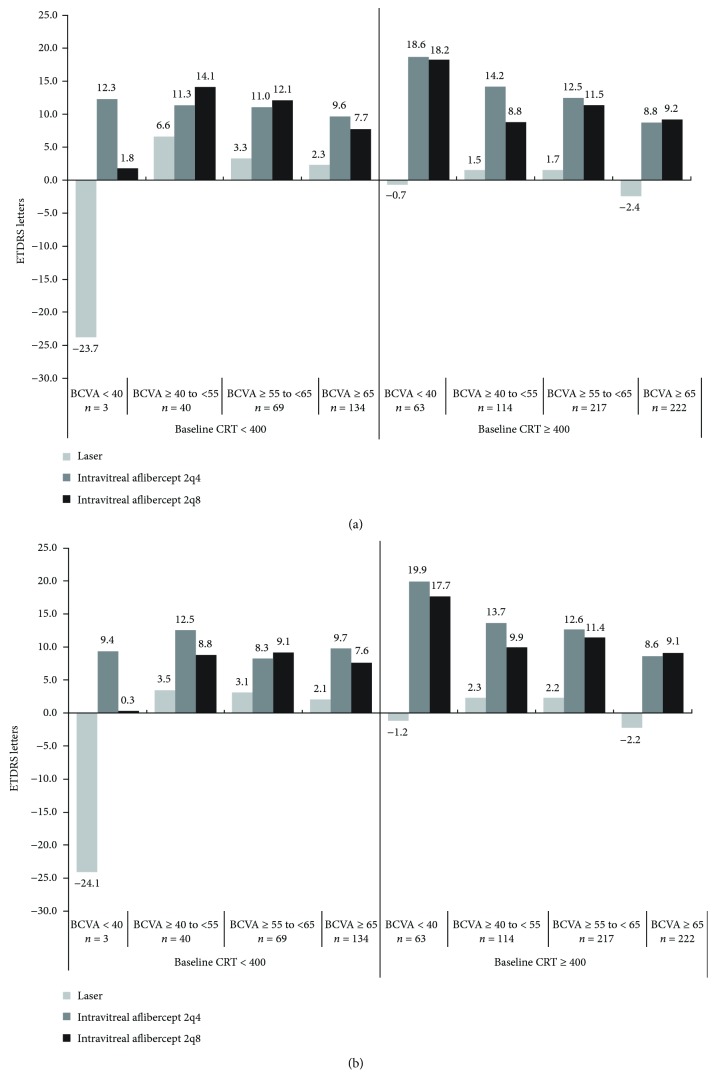
Least squares mean change in BCVA by baseline central retinal thickness and BCVA subgroups, adjusted for baseline BCVA at (a) week 52 and (b) week 100. BCVA: best-corrected visual acuity; ETDRS: Early Treatment Diabetic Retinopathy Study.

When the baseline CRT subgroups were further subdivided based on baseline BCVA, the benefits of intravitreal aflibercept over laser control were observed in all subgroups, even in those with thinner retinas at baseline, especially when baseline BCVA was impaired (Figure 2).

## 4. Discussion

The aim of these analyses was to evaluate the impact of baseline CRT on outcomes in patients enrolled in the VIVID-DME and VISTA-DME studies. Compared with laser control, visual and anatomical improvements were greater in the intravitreal aflibercept groups, regardless of the baseline CRT subgroups or the baseline CRT + baseline BCVA subgroups.

In studies of ranibizumab, the treatment effect appeared to be numerically or marginally greater in patients with thicker retinas at baseline, possibly due to the fact that patients with thicker retinas generally have worse visual acuity. In a subanalysis of the RESTORE study, patients were stratified by baseline CRT (<300 *μ*m, 300–400 *μ*m, and >400 *μ*m). Among patients treated with ranibizumab (either in monotherapy or in combination with laser), greater gains in BCVA were achieved in patients with higher baseline CRT [14]. Similarly, among ranibizumab-treated patients in the Protocol I study, patients with higher baseline central subfield thickness (CST; ≥400 *μ*m) achieved greater visual gains (mean gain of 11 letters compared with seven letters in those with baseline CST < 400 *μ*m) [15]. It should be noted, however, that these analyses did not adjust for baseline BCVA. Since the eyes with thicker retinas tend to have worse visual acuity, baseline BCVA is likely to be a confounding factor in such analyses. The http://DRCR.net conducted several subanalyses of the Protocol T data to evaluate the interaction between the treatment effect and the retinal thickness. When both visual acuity and CST and the interactions with treatment were included in the model of mean change in visual acuity, the treatment effect still varied according to the initial visual acuity (*P* = 0.006 for interaction with visual acuity), but there was less confidence in the interaction with CST (*P* = 0.11; *P* = 0.82 for the three-way interaction of visual acuity, CST, and treatment group). The results of these post hoc analyses supported the finding that baseline visual acuity is more effective than baseline CST for explaining the differences in visual outcomes across the three anti-VEGF agents at 1 year [20].

Analyses of ranibizumab data as previously described led to the NICE recommendation to limit payment for ranibizumab and, subsequently, for intravitreal aflibercept to patients with retinal thickness ≥ 400 *μ*m. In the present analyses, patients with thinner retinas at baseline (CRT < 400 *μ*m) demonstrated substantial improvements with intravitreal aflibercept over 52 and 100 weeks, and intravitreal aflibercept was superior to laser control for all visual acuity gain endpoints, regardless of baseline CRT when adjustments were made for baseline BCVA. Benefits also appear to be greater with intravitreal aflibercept than with laser control for a variety of endpoints, including visual acuity gains within all tested subcategories of baseline BCVA. While it might be expected that the eyes with thinner retinas and better vision at baseline would have limited room for improvement, in the subgroup with CRT < 400 *μ*m and BCVA ≥ 65 letters, patients receiving intravitreal aflibercept gained 7.6 (2q4 group) and 9.6 (2q8 group) letters in vision over 100 weeks of treatment, whereas those in the same subgroup of CRT and BCVA treated with laser control only gained 2.1 letters. In the natural history of DME, the functionality of the different intraretinal layers is affected by the duration of disease. A long duration of disease is associated with anatomical and functional damage [21] and decreases in the visual function-associated and health-related quality of life [22]; therefore, early treatment for DME is recommended [23]. Taken together, these observations suggest that using a clinical CRT cut-off of 400 *μ*m may unduly limit appropriate treatment options available to patients, although it should be acknowledged that organizations such as NICE also take nonclinical factors such as economics into account when formulating their decisions. When examining CRT as a continuous variable (data not shown), the analysis indicated that, when adjusted for baseline visual acuity, the mean letter gains in visual acuity increased as the baseline CRT increased with intravitreal aflibercept compared with laser. However, in spite of this relationship, no statistical evidence was uncovered in these analyses to support such differences in a binary analysis of the baseline CRT < 400 and ≥400 *μ*m subgroups. Further research is needed to elucidate this finding, as well as to confirm whether a valid CRT cut-off (if any) can be further identified.

Overall, across all subgroups assessed (i.e., based on baseline CRT alone or both baseline CRT and BCVA), there were no substantial differences in outcomes between the 2q4 and 2q8 dosing regimens. This observation further supports the argument that similar efficacy can be achieved with fewer injections using intravitreal aflibercept 2.0 mg in a q8 dosing regimen. Furthermore, consistent benefits in the DRSS score also indicate the effect of intravitreal aflibercept not only on DME but also on the underlying DR. As confirmed by previous publications [9–11], both intravitreal aflibercept treatment regimens were well tolerated with a favorable safety profile.

Strengths of VIVID-DME and VISTA-DME include the randomized design, fixed dosing, and strict protocols. However, this article reports findings from exploratory post hoc analyses and may be subject to bias. Further prospective research is needed to confirm the current findings. Future studies could compare treated and untreated patients within appropriate subgroups based on baseline CRT.

In conclusion, these post hoc exploratory analyses through week 100 of VIVID-DME and VISTA-DME demonstrated the benefits of intravitreal aflibercept over laser regardless of baseline CRT. While there is some evidence that baseline CRT seems to have an impact on adjusted mean gains in visual acuity, statistical analyses did not support a binary cut-off at 400 *μ*m as a meaningful one for the treatment effect of intravitreal aflibercept on mean BCVA gains. Given the benefits seen in the eyes with baseline CRT < 400 *μ*m, a reevaluation of the NICE restriction on prescribing intravitreal aflibercept treatment to patients with CRT < 400 *μ*m may be warranted.

## Figures and Tables

**Table 1:** Baseline demographics of patient subgroups with baseline central retinal thickness < 400 *μ*m or ≥400 *μ*m (integrated data from VIVID-DME and VISTA-DME).

	CRT < 400 *μ*m			CRT ≥ 400 *μ*m		
	Laser control (*n* = 78)	2q4 (*n* = 90)	2q8 (*n* = 78)	Laser control (*n* = 208)	2q4 (*n* = 200)	2q8 (*n* = 208)
Age (y), mean (SD)	63.1 (9.2)	61.5 (9.2)	62.6 (8.2)	62.5 (8.5)	62.7 (10.4)	64.0 (8.8)
Sex (female)	36 (46.2)	42 (46.7)	35 (44.9)	87 (41.8)	78 (39.0)	85 (40.9)
Race						
White	69 (88.5)	74 (82.2)	67 (85.9)	168 (80.8)	163 (81.5)	164 (78.8)
Other/not reported	9 (11.5)	16 (17.8)	11 (14.1)	40 (19.2)	37 (18.5)	44 (21.2)
HbA1c, mean (SD)	7.96 (1.94)	8.08 (1.55)	7.98 (1.64)	7.50 (1.28)	7.77 (1.56)	7.74 (1.44)
Duration of diabetes (y), mean (SD)	17.7 (10.1)	17.2 (10.3)	15.7 (10.0)	15.5 (9.6)	14.9 (9.4)	16.5 (10.9)
BCVA (letters), mean (SD)	63.5 (8.2)	63.1 (8.6)	63.0 (8.3)	59.0 (11.4)	58.3 (11.3)	57.7 (11.6)
<40 letters	1 (1.3)	1 (1.1)	1 (1.3)	21 (10.1)	19 (9.5)	23 (11.1)
≥40 to <55 letters	11 (14.1)	19 (21.1)	10 (12.8)	35 (16.8)	38 (19.0)	41 (19.7)
≥55–65 letters	19 (24.4)	20 (22.2)	30 (38.5)	75 (36.1)	70 (35.0)	72 (34.6)
≥65 letters	47 (60.3)	50 (55.6)	37 (47.4)	77 (37.0)	73 (36.5)	72 (34.6)
CRT (*μ*m), mean (SD)	338.8 (41.3)	343.2 (36.1)	337.9 (38.9)	568.8 (132.2)	555.1 (131.5)	551.9 (135.9)
DRSS score						
≤43	29 (37.2)	33 (36.7)	32 (41.0)	79 (38.0)	63 (31.5)	65 (31.3)
47	19 (24.4)	17 (18.9)	14 (17.9)	31 (14.9)	27 (13.5)	45 (21.6)
≥53	30 (38.5)	40 (44.4)	32 (41.0)	98 (47.1)	109 (54.5)	98 (47.1)
BMI (kg/m^2^), mean	31.48	30.04	31.72	30.02	30.97	30.02

Values are *n* (%) unless otherwise noted. 2q4: 2 mg every 4 weeks; 2q8: 2 mg every 8 weeks; BCVA: best-corrected visual acuity; BMI: body mass index; CRT: central retinal thickness; DRSS: diabetic retinopathy severity scale; HbA1c: haemoglobin A1c; SD: standard deviation.

**(a):** 

				2q4 versus laser control		2q8 versus laser control	
	Laser control (*n* = 78)	2q4 (*n* = 90)	2q8 (*n* = 78)	Difference (95% CI)^†^	*P* value^†^	Difference (95% CI) ^†^	*P* value^†^
Baseline CRT < 400 *μ*m							
LS (SE) mean change in BCVA (ETDRS letters)^∗^	3.4 (1.1)	10.8 (1.1)	10.5 (1.1)	7.3 (4.3–10.3)	<0.0001	7.1 (3.9-10.2)	<0.0001
LS (SE) mean change in CRT (*μ*m)^∗^	−111.9 (13.5)	−181.2 (12.7)	−191.2 (13.5)	−69.3 (−103.0 to −35.7)	<0.0001	−79.3 (−114.0 to −44.6)	<0.0001
Gain ≥ 10 letters	20 (25.6)	46 (51.1)	42 (53.8)	26.2 (11.8–40.6)	<0.01	28.0 (13.1–42.9)	<0.01
Gain ≥ 15 letters	8 (10.3)	27 (30.0)	17 (21.8)	20.2 (8.1–32.2)	<0.01	11.1 (−0.4 to 22.6)	0.06
Loss ≥ 10 letters	6 (7.7)	0 (0)	2 (2.6)	−11.5 (−20.3 to −2.8)	<0.01	−4.9 (−11.5 to 1.8)	0.15
Loss ≥ 15 letters	3 (3.8)	0 (0)	1 (1.3)	−5.8 (−12.2 to 0.6)	0.08	−3.7 (−11.3 to 3.9)	0.34
≥2-step DRSS improvement	9 (15.0)	22 (31.9)	18 (29.5)	17.9 (3.9–32.0)	0.01	14.8 (0.3–29.4)	0.05

**(b):** 

				2q4 versus laser control		2q8 versus laser control	
	Laser control (*n* = 208)	2q4 (*n* = 200)	2q8 (*n* = 208)	Difference (95% CI)^†^	*P* value^†^	Difference (95% CI)^†^	*P* value^†^
Baseline CRT ≥ 400 *μ*m							
LS (SE) mean change in BCVA (ETDRS letters)^∗^	−0.2 (0.7)	11.9 (0.7)	10.7 (0.7)	12.1 (10.2–14.1)	<0.0001	10.9 (9.0–12.8)	<0.0001
LS (SE) mean change in CRT (*μ*m)^∗^	−43.7	−200.2	−188.0	−156.4 (−177.9 to −135.0)	<0.0001	−144.3 (−165.6 to −123.0)	<0.0001
Gain ≥ 10 letters	44 (21.2)	128 (64.0)	118 (56.7)	42.8 (34.1–51.5)	<0.01	35.6 (26.8–44.3)	<0.01
Gain ≥ 15 letters	16 (7.7)	81 (40.5)	75 (36.1)	32.6 (24.9–40.4)	<0.01	28.4 (20.9–35.9)	<0.01
Loss ≥ 10 letters	41 (19.7)	3 (1.5)	3 (1.4)	−18.2 (−23.9 to −12.5)	<0.01	−18.3 (−23.9 to −12.6)	<0.01
Loss ≥ 15 letters	25 (12.0)	2 (1.0)	0 (0)	−11.0 (−15.6 to −6.3)	<0.01	−12.0 (−16.4 to −7.6)	<0.01
≥2-step DRSS improvement	19 (11.9)	57 (37.0)	49 (30.2)	24.9 (15.8–34.1)	<0.01	18.4 (9.7–27.1)	<0.01

Values are *n* (%) unless otherwise noted. ^∗^Adjusted for baseline BCVA. ^†^For continuous outcomes, 95% CIs and *P* values were calculated based on an analysis of a covariance model with baseline measurement, dichotomized baseline CRT (<400 *μ*m or ≥400 *μ*m), treatment group, study, and interaction between dichotomized baseline CRT, and treatment as the fixed effect. For binary outcomes, 95% CIs were calculated based on Mantel-Haenszel CI adjusted by study for each baseline CRT subgroup. 2q4: 2 mg every 4 weeks; 2q8: 2 mg every 8 weeks; BCVA: best-corrected visual acuity; CI: confidence interval; CRT: central retinal thickness; DRSS: diabetic retinopathy severity scale; ETDRS: Early Treatment Diabetic Retinopathy Study; LS: least squares; SE: standard error.

**(a):** 

				2q4 versus laser control		2q8 versus laser control	
	Laser control (*n* = 78)	2q4 (*n* = 90)	2q8 (*n* = 78)	Difference (95% CI)^†^	*P* value^†^	Difference (95% CI)^†^	*P* value^†^
Baseline CRT < 400 *μ*m							
LS (SE) mean change in BCVA (ETDRS letters)^∗^	2.9 (1.4)	10.4 (1.3)	8.5 (1.4)	7.5 (3.9–11.2)	<0.0001	5.7 (1.9–9.4)	0.0032
LS (SE) mean change in CRT (*μ*m)^∗^	−114.7 (14.9)	−182.5 (13.9)	−197.1 (14.8)	−67.8 (−104.6 to −31.0)	0.0003	−82.4 (−120.4 to −44.4)	<0.0001
Gain ≥ 10 letters	19 (24.4)	50 (55.6)	37 (47.4)	32.1 (17.9–46.3)	<0.01	23.3 (8.5–38.1)	<0.01
Gain ≥ 15 letters	7 (9.0)	30 (33.3)	17 (21.8)	25.5 (13.6–37.4)	<0.01	12.9 (1.6–24.2)	0.02
Loss ≥ 10 letters	7 (9.0)	3 (3.3)	4 (5.1)	−5.1 (−12.3 to 2.2)	0.17	−3.7 (−11.7 to 4.2)	0.36
Loss ≥ 15 letters	4 (5.1)	2 (2.2)	2 (2.6)	−2.5 (−8.5 to 3.5)	0.42	−2.5 (−8.6 to 3.5)	0.41
≥2-step DRSS improvement	11 (17.5)	21 (29.6)	23 (35.9)	14.2 (−0.2 to 28.6)	0.05	18.5 (3.2 to 33.7)	0.02

**(b):** 

				2q4 versus laser control		2q8 versus laser control	
	Laser control (*n* = 208)	2q4 (*n* = 200)	2q8 (*n* = 208)	Difference (95% CI)^†^	*P* value^†^	Difference (95% CI)^†^	*P* value^†^
Baseline CRT ≥ 400 *μ*m							
LS (SE) mean change in BCVA (ETDRS letters)^∗^	0.1 (0.8)	11.9 (0.9)	10.8 (0.8)	11.8 (9.5–14.2)	<0.0001	10.7 (8.4–13.0)	<0.0001
LS (SE) mean change in CRT (*μ*m)^∗^	−63.8 (8.8)	−215.5 (8.7)	−194.4 (8.5)	−151.7 (−175.1 to −128.3)	<0.0001	−130.5 (−153.7 to −107.4)	<0.0001
Gain ≥ 10 letters	57 (27.4)	127 (63.5)	120 (57.7)	36.0 (26.9–45.0)	<0.01	30.2 (21.2–39.3)	<0.01
Gain ≥ 15 letters	29 (13.9)	81 (40.5)	75 (36.1)	26.6 (18.3–35.0)	<0.01	22.1 (14.0–30.2)	<0.01
Loss ≥ 10 letters	48 (23.1)	7 (3.5)	3 (1.4)	−19.6 (−25.9 to −13.3)	<0.01	−21.6 (−27.6 to −15.7)	<0.01
Loss ≥ 15 letters	28 (13.5)	6 (3.0)	1 (0.5)	−10.4 (−15.6 to −5.2)	<0.01	−13.0 (−17.7 to −8.2)	<0.01
≥2-step DRSS improvement	20 (12.3)	60 (38.7)	61 (37.4)	26.0 (16.7–35.3)	<0.01	25.2 (16.2–34.1)	<0.01

Values are *n* (%) unless otherwise noted. ^∗^Adjusted for baseline BCVA. ^†^For continuous outcomes, 95% CIs and *P* values were calculated based on an analysis of a covariance model with baseline measurement, dichotomized baseline CRT (<400 *μ*m or ≥400 *μ*m), treatment group, study, and interaction between dichotomized baseline CRT, and treatment as the fixed effect. For binary outcomes, 95% CIs were calculated based on Mantel-Haenszel CI adjusted by study for each baseline CRT subgroup. 2q4: 2 mg every 4 weeks; 2q8: 2 mg every 8 weeks; BCVA: best-corrected visual acuity; CI: confidence interval; CRT: central retinal thickness; DRSS: diabetic retinopathy severity scale; ETDRS: Early Treatment Diabetic Retinopathy Study; LS: least squares; SE: standard error.
